# Factors associated with emergency admission for people dying from cancer in Northern Ireland: an observational data linkage study

**DOI:** 10.1186/s12913-023-10228-w

**Published:** 2023-10-31

**Authors:** H. Mitchell, V. Cairnduff, S. O’Hare, L. Simpson, R. White, AT Gavin

**Affiliations:** 1 Centre for Public Health, Northern Ireland Cancer Registry, Queen’s University Belfast, Belfast, Northern Ireland, United Kingdom; 2 Macmillan Cancer Support, London, England, United Kingdom; 3 Centre for Public Health, Queen’s University Belfast, Belfast, Northern Ireland, United Kingdom

**Keywords:** Emergency admissions, Cancer, End of life care, Preferred place of death

## Abstract

**Background:**

Many people living with cancer are admitted as an emergency, some just prior to diagnosis and others in their last year of life. Factors associated with accessing emergency care for people dying of cancer are complex and not well understood. This can make it difficult to have the resources and staffing in place to best care for individuals in their last year of life and their families.

**Methods:**

This study uses routinely collected administrative data from people who died of cancer in N. Ireland (NI) during 2015 and explores how personal characteristics (e.g., gender, age) and disease related factors (e.g., tumour site, cancer stage at initial diagnosis) were associated with having an emergency admission to hospital in the last year and the last 28 days of their lives, using multivariate logistic regression.

**Results:**

Almost three in four people had at least one emergency admission in the last year of life, and over one in three had an emergency admission the last 28 days of life. Patterns were similar for both time outcomes with males, people with haematological, lung or brain cancers, younger persons, those diagnosed with late-stage cancer, and people diagnosed close to time of death, being significantly more likely to have an emergency admission. While there was no significant association between deprivation and emergency admission rates, those living in urban areas were more likely to have an emergency admission in their last month of life compared to rural dwellers. Late diagnosis was evident with 538 people (12.8% of all deaths from cancer) being diagnosed within one month of death and 1242 (29%) within 3 months of death.

**Conclusion:**

The high level of emergency admissions points to gaps in routine end-of-life care, and the need for additional training for hospital staff including frontline emergency department (ED) staff who are often the ‘gatekeepers’ to emergency inpatient care for people living with cancer. The levels of late diagnosis indicate a need for increased population awareness of cancer symptoms and system change to promote earlier diagnosis.

**Supplementary Information:**

The online version contains supplementary material available at 10.1186/s12913-023-10228-w.

## Introduction

Choice about the place of care and death is an important component of end-of-life care and there is increasing interest in the timing and location of this care for people with cancer. In 2010 ‘Living Matters Dying Matters: A palliative and end of life care strategy for adults in Northern Ireland (NI) ’ [1] was published. This Strategy emphasised several features of good palliative care, which include the need to: support life and view dying as a normal process; provide relief from pain and other symptoms; integrate all aspects of patient care; offer holistic support to allow people to live as actively as possible until death; use a team approach; help family cope both during the illness and into bereavement; and neither to accelerate nor postpone death [1].

Several studies have shown that the majority of people dying of cancer prefer to be cared for and die at home [2–5], and the most important aspects of care during this time are adequate control of symptoms, time spent with loved ones, and access to emotional and spiritual support when needed [6–8]. However, in order to facilitate these preferences for care, factors such as access to medical care, control of symptoms and family support must be carefully considered [9].

A systematic review [4] of 210 studies investigating people with terminal cancer and changes in their preferred place of death (PPD) over time concluded that 75% of the studies showed that over half of people preferred to die at home. Studies [8, 10, 11] have also shown that the majority preferred to limit ‘aggressive’ care, concentrating instead on achieving good symptom control and planning for their changing care needs associated with disease progression. Several studies [12–15] have shown a relationship between increased use of aggressive treatments towards the end of life and poorer patient and family or carer outcomes - such as prolonged pain, overall dissatisfaction with care and more than three times the odds of psychiatric illness in bereaved relatives.

Increased use of acute hospital care, and in particular emergency admissions towards the end of life, may indicate gaps in routine end-of-life care and/or a lack of recognition that a person is approaching the end of their life and requires advance care planning and palliative care support. However, many symptoms experienced at the end of life do require specialist palliative care and sometimes this can only be provided in hospital. A recent systematic review [16] of 42 studies investigated effectiveness and cost-effectiveness of hospital‐based specialist palliative care (HSPC) compared to usual care for adults with advanced illness found that those who accessed HSPC experienced higher health-related quality of life and lower symptom burden compared to those who were treated with usual care after admission.

Determining which hospital visits represent necessary care for the adequate relief of symptoms is very challenging when faced with people living with a terminal illness and access to specialist services is important to ensure quality of life is maintained as far as possible. There is currently limited population-based evidence on the proportion of individuals with cancer having an emergency admission in the last year of life as well as the factors associated an emergency admission.

The aim of this study was to estimate the relative strength of association of several factors in people dying of cancer having an emergency admission to hospital in **(I)** the last year of life and **(II)** the last 28 days of life.

## Methodology

Data on demographic and disease characteristics for people who died from cancer in NI in 2015 was extracted from the Northern Ireland Cancer Registry (NICR) database, linked with death registration data obtained from the General Register Office NI and with emergency admission episodes from the Patient Administration System (PAS), which records hospital admission episodes in NI, from 1st January 2014 and 31st December 2015. The data were anonymised for analysis. Emergency admission episodes are defined an admission to an acute hospital ward that has received the clinical code for emergency admission within PAS. These admissions may have been directly due to cancer and/or cancer treatment, a comorbid condition or cancer may be found incidentally during an emergency admission for another reason. Attendances to the Emergency Department (ED) that were not subsequently admitted to a ward are not captured in this analysis.

A small number of people (n = 92) who died of cancer in 2015 were excluded from the study due to a lack of clinical information around their time of death (n = 54), or because they were extra-regional at the time of their cancer diagnosis (n = 38) and therefore information was not available within the NICR database. There were no significant differences between those included and excluded from the final analysis regarding sex (p = .872), age at time of death (p = .139) or deprivation quintile (p = .392).

The variables used in the analysis included the following categories: *Personal demographics*: sex, age at time of death, urban/rural location and deprivation quintile (both based on postcode of residence at diagnosis); *Disease characteristics*: tumour type, stage at diagnosis and time from diagnosis to death; *Emergency admission*: number of admissions in last year; and *Place of death*.

Two separate binary outcome variables were created for the analysis:

(I) “people who had at least one emergency admission in last year of life (LYOL)” (Yes/No), and.
(II) “people who had at least one emergency admission in last 28 days of life (LMOL)” (Yes/No).

### Statistical analysis

This study used descriptive statistics (e.g., two and three-way Pearson’s chi-squared tests) to explore if demographic, disease, and environmental characteristics were significantly associated (at the 95% confidence interval) with the outcome variables.

Where relationships were significant, the strength of the association was described using *Phi* and *Cramer’s V* and adjusted residuals were used to describe the direction of the association (with significant differences within variables measured at the p < .05 level).

A multivariate logistic regression was performed to obtain a model that could accurately predict which people were most likely to have an emergency admission in the last month of life. Full reporting of test statistics is included in Tables 1 and 2 and Supplementary Table 1.

**Table 1:** Demographic and Disease Characteristic variables for people living with cancer by emergency admission rates in the last year, and last 28 days, of life^1^

Variables	Categories (N for cohort)	% of cohort with an emergency admission in the last year of life	% of cohort with an emergency admission in last 28 days of life
Sex	Male (N = 2306)	75.5% ↑	39.2% ↑
	Female (N = 1953)	72.2% ↓	33.4% ↓
	All people (N = 4259)	74.0%	36.5%
	Test result^2^ Strength of association (SOA): Interpretation of SOA:	Χ^2^ (1, N = 4,259) = 5.83 p = .016 Φ= 0.037 Very weak	X^2^ (1, N = 4259) = 15.44. p < .001 Φ = 0.060 Very weak
Age at time of death (in years)	0 to 39 (N = 66)	75.8%	37.9%
	40 to 49 (N = 146)	81.5%	43.8%
	50 to 59 (N = 370)	83.5% ↑	42.2% ↑
	60 to 69 (N = 911)	79.9% ↑	42.7% ↑
	70 to 79 (N = 1302)	74.4%	34.3% ↓
	80 to 89 (N = 1149)	68.8% ↓	33.6% ↓
	90 and over (N = 315)	58.4% ↓	28.3% ↓
	Test result: Strength of association (SOA): Interpretation of SOA:	Χ^2^ (6, N = 4,259) = 94.03. p < .001 Φc = 0.149 Weak	Χ^2^ (6, N = 4,259) = 39.72. p < .001 Φc = 0.097 Very weak
Deprivation based on postcode of residence at time of diagnosis.	Quintile 1 least deprived (N = 822)	72.9%	33.9%
	Quintile 2 (N = 869)	74.5%	36.1%
	Quintile 3 (N = 812)	75.0%	38.4%
	Quintile 4 (N = 824)	73.3%	37.1%
	Quintile 5 most deprived (N = 923)	74.3%	37.1%
	Unknown (N = 9)	55.6%	33.3%
	Test result:	X^2^ (4, N = 4250) = 1.31. p = .858	X^2^ (4, N = 4250) = 3.93. p = .416
Location in which person lived (at diagnosis)	Urban (N = 2857)	74.8%	37.6% ↑
	Rural (N = 1393)	72.3%	34.4% ↓
	Unknown (N = 9)	55.6%	33.3%
	Test result: Strength of association (SOA): Interpretation of SOA:	Χ^2^ (1, N = 4,250) = 3.149 p = .094 n/a n/a	X^2^ (1, N = 4250) = 4.15. p = .042 Φ = 0.031 Very weak
Most recent tumour site	Brain & CNS (N = 104)	79.8%	28.8%
	Breast (N = 277)	70.0%	33.2%
	Colorectal & Anus (N = 492)	74.0%	32.3% ↓
	Haematological (N = 367)	80.4% ↑	42.0% ↑
	Head & Neck (N = 137)	64.3% ↓	28.5% ↓
	Lung & Mesothelioma (N = 1051)	78.1%↑	42.8% ↑
	Other digestive (N = 791)	75.6%	38.9%
	Urinary (N = 234)	73.5%	37.2%
	Prostate (N = 254)	74.4%	35.0%
	Unknown primary (N = 162)	64.2% ↓	31.5%
	Other (N = 390)	62.1% ↓	24.9% ↓
	Test result: Strength of association (SOA): Interpretation of SOA:	Χ^2^ (10, N = 4,259) = 65.97. p < .001 Φc = 0.124 Weak	Χ^2^ (10, N = 4,259) = 61.06. p < .001 Φc = 0.120 Weak
Variables	Categories (N for cohort)	% of cohort with an emergency admission in the last year of life	% of cohort with an emergency admission in last 28 days of life
Stage at Diagnosis	Stage I (N = 236)	67.4% ↓	31.8%
	Stage II (N = 370)	70.5%	31.9%
	Stage III (N = 701)	77.5% ↑	36.5%
	Stage IV (N = 1508)	79.7% ↑	40.8% ↑
	Unknown (N = 1444)	68.2% ↓	34.1% ↓
	Test result: Strength of association (SOA): Interpretation of SOA:	Χ^2^ (4, N = 4,259) = 62.66. p < .001 Φc = 0.121 Weak	Χ^2^ (4, N = 4,259) = 21.25. p < .001 Φc = 0.071 Very weak
Time from Diagnosis of most recent cancer to Death	0 to 28 days (N = 538)	71.6%	61.0% ↑
	1 to 3 months (N = 704)	74.4%	32.0% ↓
	3 to 6 months (N = 495)	77.0%	29.7% ↓
	6 to 9 months (N = 375)	82.1% ↑	34.1%
	9 to 12 months (N = 283)	78.8%	33.6%
	1–2 years (N = 566)	75.1%	36.2%
	2–3 years (N = 403)	75.2%	35.2%
	3–4 years (N = 215)	69.8%	37.2%
	4–5 years (N = 139)	67.6%	33.8%
	5–10 years (N = 321)	64.1% ↓	29.1% ↓
	10 years+ (N = 215)	68.8%	29.8% ↓
	Unknown (N = 3)	66.7%	33.3%
	Test result: Strength of association (SOA): Interpretation of SOA:	Χ^2^ (10, N = 4,256) = 45.37. p < .001 Φc = 0.103 Weak	Χ^2^ (10, N = 4,256) = 169.58. p < .001 Φc = 0.200 Moderate
Place of Death	Home (N = 1498)	64.9% ↓	18.6% ↓
	Hospital (N = 1768)	85.0% ↑	63.3% ↑
	Hospice (N = 457)	74.8%	19.3% ↓
	Nursing Home (N = 446)	60.5% ↓	12.3% ↓
	Other (N = 90)	70.0%	16.7%
	Test result: Strength of association (SOA): Interpretation of SOA:	Χ^2^ (4, N = 4,259) = 218.79. p < .001 Φc = 0.227 Moderate	Χ^2^ (4, N = 4,259) = 939.98. p < .001 Φc = 0.470 Very strong
Number of Emergency admissions in last year of life	0 (N = 1109)	26%	
	1 (N = 1575)	37%	
	2 (N = 865)	20.3%	
	3 (N = 376)	8.8%	
	4 (N = 178)	4.2%	
	5+ (N = 156)	3.7%	

↑denotes categories with significantly higher emergency admissions than expected, and ↓ denotes categories with significantly lower emergency admissions than expected, both results are at the p < .05 level

**Table 2:** Emergency admission rates, in the last year of life, for people who died of cancer in 2015, by tumour site

Tumour site	Emergency admission rates in the last year of life		
	No emergency admissions	1–2 emergency admissions	3 or more emergency admissions
Prostate (n = 254)	25.6%	53.1%	21.3% ↑
Haematological (n = 367)	19.6% ↓	59.9%	20.4% ↑
Colorectal & Anus (n = 492)	26.0%	53.9%	20.1% ↑
Urinary (n = 234)	26.5%	54.3%	19.2%
Lung & Mesothelioma (n = 1051)	21.9% ↓	62.0% ↑	16.1%
Other digestive (n = 791)	24.4%	59.5%	16.1%
Breast (n = 277)	30.0%	54.5%	15.5%
Other (n = 390)	37.9% ↑	47.7% ↓	14.4%
Head & Neck (n = 137)	35.8% ↑	51.1%	13.1%
Brain & CNS (n = 104)	20.2%	68.3% ↑	11.5%
Unknown primary (n = 162)	35.8% ↑	56.8%	7.4% ↓
Totals for all (n = 4259)	26.0%	57.3%	16.7%
Test result: Strength of association (SOA): Interpretation of SOA:	X^2^ (20, N = 4259) = 89.62. p < .001 Φc = 0.103 Weak		

## Results

Records of 4259 people who died of cancer in 2015 were examined. Almost three quarters (74%) of all people who died from cancer in 2015 had a least one emergency admission in their last year of life (LYOL) and more than a third (36.5%) had at least one emergency admission in their last month of life (LMOL).

Emergency admissions were significantly more common for males in both the LYOL (75.5%) and the LMOL (39.2%), people aged 50–59 and 60–69, in both the LYOL (83.5% and 79.9%) and in the LMOL (42.2% and 42.7%), those diagnosed with haematological cancers (80.4% LYOL, 42% LMOL), lung/mesothelioma cancers (78.1% LYOL, 42.8% LMOL) or brain and CNS cancers (79.8% LYOL) and males with colorectal and anus cancers (78.4% LYOL) and males with lung and mesothelioma cancers (47.8% LMOL). Other characteristics which were associated with a significantly increased likelihood of having at least one emergency admissions were having a stage IV cancer (79.9% LYOL, 40.8% LMOL), being male and diagnosed at Stage IV (44.9% LMOL), those aged 60–69 diagnosed at Stage IV (45.5% LMOL), those aged under 40 diagnosed at Stage II (100% LMOL), and those aged 50–59 diagnosed at Stage III (53.9% LMOL). Additionally, people diagnosed 6–9 months prior to death (82.1% LYOL) and people diagnosed in the last month of life (61% LMOL) were also at significantly higher likelihood of an emergency admission.

Having at least one emergency admissions was significantly less common for people aged 80–89 (68.8% LYOL, 33.6% LMOL) and 90 and over (58.4% LYOL, 28.3% LMOL), those with head and neck (64.2% LYOL, 28.5% LMOL), unknown primary (64.2% LYOL), other cancers (62.1% LYOL, 24.9% LMOL) and colorectal and anus cancers (32.3% LMOL). Other characteristics which were associated with a significantly reduced likelihood of having at least one emergency admissions were having an “Unknown” stage at diagnosis (68.2% LYOL, 34.1% LMOL) and Stage I cancers (67.4% LYOL), being aged 90 or over diagnosed at Stage II (8% LMOL), being aged 70–79 with a Stage IV cancer (37.1% LMOL) or 80–89 (35.2% LMOL) and those diagnosed between 5 and 10 years ago (64.1% LYOL).

### Location in which people dying of cancer lived

People living in rural areas (72.3%) and people from urban areas (74.8%) accessed emergency admissions in their LYOL at similar rates. However, people from urban areas (37.6%) were significantly more likely than people from rural areas (34.4%) to have an emergency admission in LMOL. There was, however, no significant association between deprivation and having an emergency admission in the LMOL or in the LYOL.

### Place of death

People who died in hospital were significantly more likely to have had an emergency admission (85% LYOL, 63% LMOL) with people who died at home (64.9% LYOL, 18.6% LMOL) or in a nursing home (60.5% LYOL, 12.3% LMOL) being significantly less likely to have had an emergency admission.

### Time from diagnosis to death

A third (33.9%) of those admitted as an emergency in the last year of life were either admitted on the same day as their cancer diagnosis (10.9%) or within the three months (23.0%) before their cancer diagnosis. This represents a quarter (24.9%) of cancer deaths in NI in 2015. People who died within 28 days from diagnosis (n = 538, 12.8% of those who died of cancer in 2015) were more likely to have had an emergency admission in LMOL (61%).

See Table 1 for more information and related test results.

While 26% of people who died of cancer in 2015 had no emergency admission in the LYOL, 37% had one emergency admission, 16.7% had three or more admissions, and 3.7% had five or more emergency admissions. Having three or more emergency admission in the LYOL was significantly more common among people living with prostate cancer (21.3%), haematological cancer (20.4%), or colorectal/anal cancers (20.1%) - see Table 2 for more information.

### Cancer site and gender

Lung and Mesothelioma cancer was the only site in which males (47.8%) and females (36.2%) experienced significant differences in emergency admission rates in the last month of life.

#### Multivariate logistic regression for emergency admissions in the last month of life

A multivariate logistic regression was conducted to better understand how personal, environmental and diseases variables (i.e., gender, age at death, cancer site, stage at diagnosis, location in which person lived, and deprivation) interacted with each other and the outcome variable “having an emergency admission in the last month of life”.

While the resulting model was a significant improvement relative to an intercept-only model, the predictive quality of the model was too low to be insightful; with only 10.3% of positive cases (i.e., people who had an emergency admission in the month of life being correctly predicted as having an emergency admission) and an overall correctly classified rate of 63.9%. Supplementary Table 1 displays more information on the multivariate logistic regression (and three-way crosstabulation results).

## Discussion

This is one of very few population-based studies in the UK to explore factors associated with emergency admissions for people living with cancer in the last year and month of life. Analysis has shown variation by tumour site, gender, age, and stage at diagnosis.

This study found that 75% of all people who died from cancer in 2015 had a least one emergency admission in their last year of life (LYOL). A recent population-based study looking at palliative chemotherapy in people living with breast cancer, found that 76% of people had an emergency admission in the last 90 days of life [17].

### Tumour site

It is likely that the variation in emergency admissions for the different tumour sites may be due to the symptoms that develop with progression of these cancers. Hui et al. [18] found that in the last month of life people with a haematological malignancy were more likely to have an emergency outpatient visit to hospital, two or more hospital admissions and an inpatient stay of more than two weeks compared to people with solid tumours. This current study found similar results, with a high proportion of patients with haematological cancers have at least one emergency admission in LYOL (80.4%) and LMOL (42%). Possible reasons for the increased admissions for those with a haematological cancer are the development of infections and a reduction in the number of mature blood cells and platelets. These symptoms can require frequent admission to hospital, invasive investigations, monitoring and therapies [19].

### Gender

The current study did find that males had higher emergency admission rates compared to females across the majority of tumour sites. However, this difference was statistically significant only for people living with colorectal and anal cancer in the last year of life and people living with lung and mesothelioma cancer in the last month of life. Although not completely comparable, a recent study in England [20] found that males were significantly more likely than females to have ‘avoidable’ emergency department attendances.

### Stage at diagnosis

This study again highlighted the problem of late diagnosis of cancer. As shown in the results, a third (33.9%) of those admitted as an emergency in the last year of life were either admitted on the same day as their cancer diagnosis (10.9%) or within the three months (23.0%) before their cancer. This indicates that emergency admissions are a key route to diagnosis for these individuals. This is comparable with international data that shows between 24% and 43% of cancers were diagnosed during an emergency presentation [21]. People diagnosed with cancer following an admission to hospital as an emergency tend to have a higher stage at diagnosis and poorer clinical outcomes than those diagnosed through all other routes including screening or GP referral, thus impacting survival.

### Age and deprivation

In this study it was found that those aged between 50 and 70 years had the most emergency admissions. It has previously been reported that the proportion of people diagnosed with cancer as the result of an emergency hospital admission is higher among those who are older, have late stage cancer [22]and live in more deprived areas [23]. However, the shape of the variation by age in this dataset is slightly different, with people aged 70 and over, in general, being less likely to have an emergency admission in the last year, and last month of life, than people aged between 40 and 69. This is similar to the findings of Bright et al. [17], who found that 83% of people aged less than 45 years old had an ED admission compared to 67% of people aged 80 years and over. This finding could be reflective of more aggressive cancers in the younger age groups or being more likely to receive treatments which may be less well tolerated in older adults.

In this study there was no relationship with deprivation and the higher admission rates in last month of life in urban vs. rural areas may reflect proximity to hospital.

### Place of death

This study found that those with at least one emergency admission in the last year of life were twice as likely to die in hospital, and even higher if admitted in last month of life when compared to those with no emergency admissions. These findings are similar to previous studies [4] that have shown that although many people would prefer to die at home, there are challenges in addressing the needs of people outside secondary care.

### Multiple admissions

In this study 37% of people had two or more emergency admission in last year of life. There is evidence to suggest that engagement with palliative care in hospital can reduce the number of readmissions [24]. In reducing the readmission rate people may be more likely die in their preferred place of death.

### Strengths and weaknesses of the study

This study illustrates the value of linkage of high quality, population based registry data [25] to hospital administrative data, a recommendation from an international study to enhance cancer surveillance both within jurisdictions and internationally and enable better understanding of variations in survival [21]. We have described, for the first time, the frequency of emergency admissions near the end of life at a population level and given insight into the characteristics of people who experience such admissions. The work did not include emergency attendances which resulted in discharge on the same day and so does not reflect the full burden on people living with cancer on Acute hospital services.

The limitations of this study include the lack of clinical data on the symptoms that led to the admission and co-morbidities at admission and therefore data presented here do not provide information to determine if the emergency admissions were potentially avoidable or the quality of care in the different settings. Bright et al. [17] found that palliative chemotherapy provided near the end of live was associated with increased unplanned admissions and death in hospital. This current study did not look at the treatments given and the timings of these treatments. A significant period of time has passed since 2015 and the healthcare landscape has altered over this time. Though this may limit the generalisability of the results, similar patterns have been observed in more recent studies [26]. To the authors’ knowledge, this is the only study of its kind at a population-level in NI.

### Future work

The NICR (Northern Ireland Cancer Registry) has recently been given permission to use additional patient data (on co-morbidities, cancer treatments etc.) and could add more high-quality data to this existing study that would help with further modelling. In more recent years we have seen the impact of COVID-19 on emergency admissions for people living with cancer [27], though it is not known the impact on those in last year of life and could differ from the rest of the cancer population.

## Conclusions

In conclusion, we have documented a high level of utilisation of emergency admission near the end of the lives of people living with cancer and provided some insight into patterns of use by gender, age, tumour site and stage of diagnosis.

Three in four people dying of cancer had an emergency admission in their last year of life, and one in three emergency admission in their last month of life with many diagnosed late in their journey. The high level for ED admissions in the last year and month of life point to the need for:

- Raising awareness among the population about cancer symptoms to reduce late diagnosis.
- Ensuring that acute oncology and palliative care expertise are available to the generalist Health Care Professionals who are assessing people living with cancer on a 24 − 7 basis including ‘out of hours’.
- Health Care Professionals to have advance care planning conversations with this cohort of people.

### Electronic supplementary material

Below is the link to the electronic supplementary material.

Supplementary Material 1

## Data Availability

The datasets generated and/or analysed during the current study are not publicly available due the limits of the ORECNI ethical approval granted to the NICR to share patient level data. Anonymised, non-patient level data can be made available from the corresponding author on reasonable request.

## Abbreviations

ED: Emergency department
NI: Northern Ireland
NICR: Northern Ireland Cancer Registry
