# Supplementary material for: Factors associated with emergency admission for people dying from cancer in Northern Ireland: an observational data linkage study
Source: BMC Health Serv Res. 2023 Oct 31;23:1184. doi: 10.1186/s12913-023-10228-w (PMC10617099; doi:10.1186/s12913-023-10228-w)
Supplement: Supplementary file 1 — Supplementary Material 1 [file 12913_2023_10228_MOESM1_ESM.docx]

**Factors associated with emergency admission for people dying from cancer in Northern Ireland: An Observational Data Linkage Study - Supplementary material**

H Mitchell^1*^, V Cairnduff^3^, S O’Hare^1^, L Simpson^1^, R, White^2^, AT Gavin^1^

^1^Northern Ireland Cancer Registry, Centre for Public Health, Queen’s University Belfast, Belfast, Northern Ireland, United Kingdom.

^2^Macmillan Cancer Support, England, United Kingdom.

^3^ Centre for Public Health, Queen’s University Belfast, Belfast, Northern Ireland, United Kingdom.

*Corresponding author – helen.mitchell@qub.ac.uk

**Supplementary Table 1:** Test results from T-test, three-way crosstabulations, and binary logistic regression

| Tests applied | Categories | For people who had an emergency admission in the last year of life | For people who had an emergency admission in the last month of life |
| --- | --- | --- | --- |
| Mean number of emergency admissions in last year of life | Male (N=2306) | Mean: 1.5 **↑**  Standard deviation: 1.43 | N/A |
|  | Female (N=1953) | Mean: 1.34 **↓**  Standard deviation: 1.35 | N/A |
|  | Test result: | t(4206.14) = -3.67,  p< .001 | N/A |
| Three-way crosstabulations | | | |
| Cancer site and gender | Categories with significant associations:  Males  Females  Test result:  Strength of association (SOA):  Interpretation of SOA: | **Site: Colorectal and Anus**  78.4% **↑**  68.9% **↓**  Χ^2^ (1, N=492) = 5.79.  p = .016  Φc= .109  Weak | **Site: Lung and Mesothelioma**  47.8% **↑**  36.2% **↓**  Χ^2^ (1, N = 1051) = 14.13. p < .001  Φc= .116  Weak |
| Cancer stage at diagnosis and gender | Categories with significant associations:  **Males**  **Females**  Test result:  Strength of association (SOA):  Interpretation of SOA: | No significant associations | **Stage IV:**  44.9% **↑**  35.3% **↓**  Χ^2^ (1, N = 1508) = 14.10.  p < .001  Φc= .097  Weak |
| Age at death and cancer stage at diagnosis | Categories with significant associations:  **Aged 90 and over**  Test result:  Strength of association (SOA):  Interpretation of SOA:  **Aged 50-59**  Test result:  Strength of association (SOA):  Interpretation of SOA:  **Aged 60-69**  **Aged 70-79**  **Aged 80-89**  Test result:  Strength of association (SOA):  Interpretation of SOA: | Not conducted | **Stage II**:  8.0% **↓**  Χ^2^ (6, N =370) = 21.05.  p = .002  Φc= .239  Moderate  **Stage III:**  53.9% **↑**  Χ^2^ (6, N =701) = 19.06.  p = .004  Φc= .165  Weak  **Stage IV:**  45.5% **↑**  37.1% **↓**  35.3% **↓**  Χ^2^ (6, N=1508) = 17.15.  p = .009  Φc= .107  Weak |
| Binary logistic regression | | | |
| Outcome = emergency admission in last month of life | Variables included: Age at death, Gender, Stage at diagnosis, Location in which person lived, Cancer site, Deprivation.  Model chi-square test:  Model goodness of fit  (Hosmer & Lemeshow test):  Sensitivity of model:  Correctly classified: | Not conducted | Χ^2^ (26, N=4250) =132.97.  p < .001  Χ^2^ (8, N=4250) = 6.79.  p = .559  10.3%  63.9% |

**Supplementary Figure 1:** Proportion of people who died from cancer in 2015 who had an emergency admission **in the last year of life**, for selected cancer sites by gender.

**Supplementary Figure 2:** Proportion of people who died from cancer in 2015 who had an emergency **in the last month of life**, for selected cancer sites by gender.

**Supplementary Figure 3:** Proportion of people who died from cancer in 2015 who had an emergency admission **in the last year of life**, by stage at diagnosis and gender.

**Supplementary Figure 4:** Proportion of people who died from cancer in 2015 who had an emergency admission **in the last month of life**, by stage at diagnosis and gender.
